# {2-[2,2-Bis(4,4-dimethyl-4,5-di­hydro-1,3-oxazol-2-yl-κ*N*)prop­yl]pyridine}­dichlorido­iron(II)

**DOI:** 10.1107/S1600536813018047

**Published:** 2013-07-06

**Authors:** Giuseppina Roviello, Angela Tuzi, Carmine Capacchione, Stefano Milione, Claudio Ferone

**Affiliations:** a Dipartimento di Ingegneria, Università di Napoli ’Parthenope’, Centro Direzionale di Napoli, Isola C4, 80143 Napoli, Italy; b Dipartimento di Scienze Chimiche, Università degli Studi di Napoli ’Federico II’, Complesso di Monte S. Angelo, Via Cinthia, 80126 Napoli, Italy; c Dipartimento di Chimica e Biologia, Università degli Studi di Salerno, Via Giovanni Paolo II 132, 84084 Fisciano (Salerno), Italy

## Abstract

The title compound,[FeCl_2_(C_18_H_25_N_3_O_2_)], has a distorted tetra­hedral Cl_2_N_2_ coordination of the Fe^II^ atom as a result of the constraints imposed by the 2-[2,2-bis­(4,4-dimethyl-4,5-di­hydro-1,3-oxazol-2-yl)prop­yl]pyridine ligand. The pyridine ring is almost perpendicular to the six-membered chelated ring containing the metal atom [dihedral angle between their mean planes = 88.5 (1)°].

## Related literature

For the analogous bis­(oxazoline)iron(II) complex, see: Ferro *et al.* (2007 ▶). For active catalysts used in atom-transfer radical polymerization (ATRP) reactions, see: Matyjaszewski & Xia (2001 ▶); Kamigaito *et al.* (2001 ▶); De Roma *et al.* (2011 ▶); Ferro *et al.* (2009 ▶). For similar salicylaldiminato complexes, see: O‘Reilly *et al.* (2003 ▶). For structural data on metal complexes, see: Li, Lamberti, Mazzeo *et al.* (2012 ▶); Li, Lamberti, Roviello *et al.* (2012 ▶); Busico *et al.* (2006 ▶); D‘Auria *et al.* (2012 ▶). For N-rich aromatic heterocycles, see: Carella *et al.* (2012 ▶), Roviello *et al.* (2012 ▶); Milione & Bertolasi (2011 ▶).
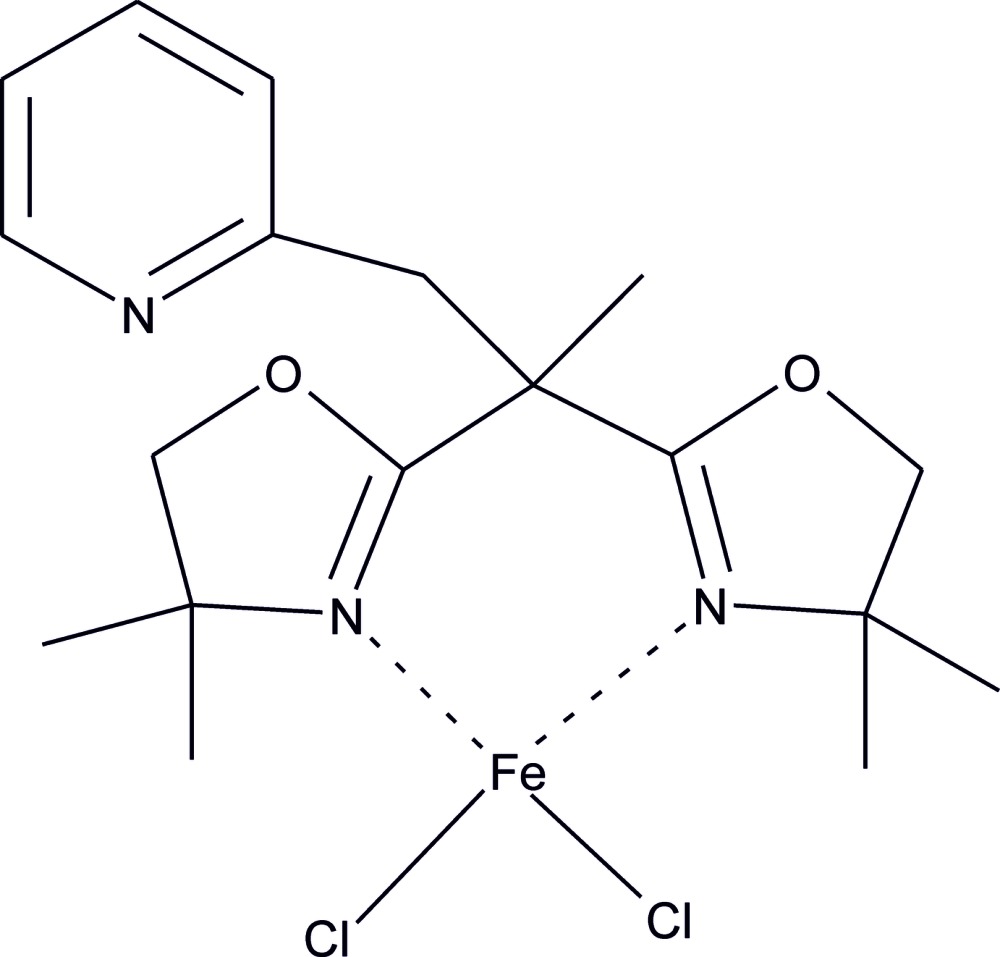


## Experimental

### 

#### Crystal data

[FeCl_2_(C_18_H_25_N_3_O_2_)]
*M* *_r_* = 442.16
Monoclinic, 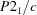

*a* = 10.102 (2) Å
*b* = 13.925 (3) Å
*c* = 14.764 (2) Å
β = 105.27 (1)°
*V* = 2003.5 (6) Å^3^
*Z* = 4
Mo *K*α radiation
μ = 1.04 mm^−1^
*T* = 173 K
0.20 × 0.10 × 0.10 mm

#### Data collection

Bruker–Nonius KappaCCD diffractometer
Absorption correction: multi-scan (*SADABS*; Bruker, 2001 ▶) *T*_min_ = 0.820, *T*_max_ = 0.903
16483 measured reflections
4578 independent reflections
3033 reflections with *I* > 2σ(*I*)
*R*_int_ = 0.054

#### Refinement

*R*[*F* ^2^ > 2σ(*F* ^2^)] = 0.041
*wR*(*F* ^2^) = 0.083
*S* = 1.01
4578 reflections
240 parameters
H-atom parameters constrained
Δρ_max_ = 0.34 e Å^−3^
Δρ_min_ = −0.30 e Å^−3^

### 

Data collection: *COLLECT* (Nonius, 1999 ▶); cell refinement: *DIRAX/LSQ* (Duisenberg *et al.*, 2000 ▶); data reduction: *EVALCCD* (Duisenberg *et al.*, 2003 ▶); program(s) used to solve structure: *SIR97* (Altomare *et al.*, 1999 ▶); program(s) used to refine structure: *SHELXL97* (Sheldrick, 2008 ▶); molecular graphics: *ORTEP-3 for Windows* (Farrugia, 2012 ▶); software used to prepare material for publication: *WinGX* (Farrugia, 2012 ▶).

## Supplementary Material

Crystal structure: contains datablock(s) global, I. DOI: 10.1107/S1600536813018047/gg2121sup1.cif

Structure factors: contains datablock(s) I. DOI: 10.1107/S1600536813018047/gg2121Isup2.hkl

Additional supplementary materials:  crystallographic information; 3D view; checkCIF report

## Figures and Tables

**Fig. 1.**
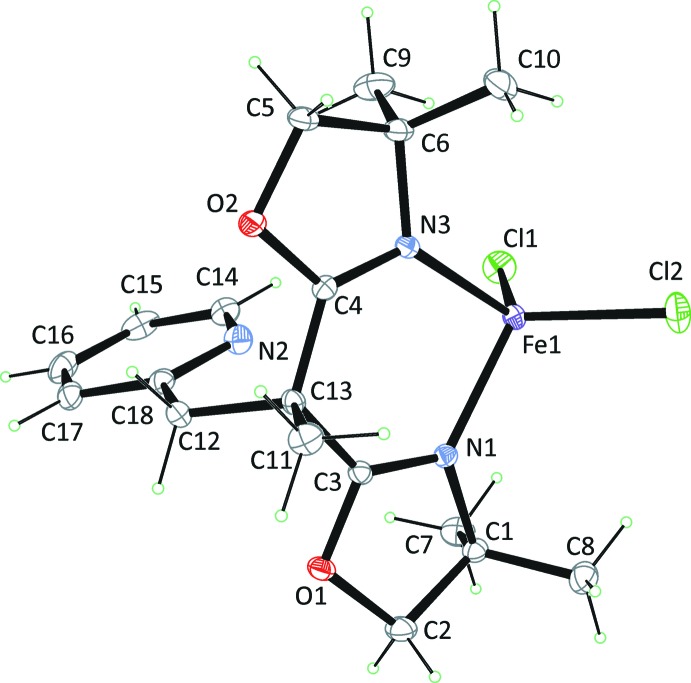
ORTEP view of the title compound. Thermal ellipsoids are drawn at 30% probability level.

## supplementary crystallographic information

### Comment

Metal complexes containing bis(oxazoline) ligands have been widely investigated in the field of homogeneous and asymmetric catalysis. This type of ligands could stabilize the pseudotetrahedral coordination environment of iron(II) affording to efficient ATRP catalyst. The complex Fe(box-py)Cl_2_ is analogous to Fe(box)Cl_2_, described in the literature (Ferro *et al.*, 2007) and has been investigated in order to obtaining an improved understanding of the factors influencing efficient ATRP catalysis in iron-based systems. The title compound Fe(box-py)Cl_2_ (box-py = 2-[2,2-Bis-(4,4-dimethyl-4,5-dihydro-oxazol-2-yl)-propyl]-pyridine), C_18_H_25_Cl_2_Fe_1_N_3_O_2_, has been used as catalyst in the framework of still not published studies on atom transfer radical polymerization (ATRP) of styrene and methylmethacrylate. The molecular structure of Fe(box-py)Cl_2_ shows a distorted tetrahedral geometry, due to the constraints imposed by the ligand (N1—Fe1—N3 = 88.85 (7)°; Cl1—Fe1—Cl2 = 113.81 (3)°). The Fe—N and Fe—Cl bond distances are similar to that found for Fe(box)Cl_2_ and for similar saliclylaldiminato complex (O`Reilly *et al.*, 2003). Finally, the heteroaromatic ring is almost perpendicular to the six-membered chelated ring (angle between their mean planes is equal to 88.5 (1)°) and shows the nitrogen atom pointing towards the coordination environment of the iron.

### Experimental

Fe(box-py)Cl_2_ was obtained by the reaction of FeCl_2_ with the ligand. Details for the synthesis will be reported in forthcoming work. Prismatic yellow crystals of the title complex were obtained by slow evaporation of a methylene chloride solution at room temperature.

### Refinement

All the H atoms were generated stereochemically and refined by the riding model with C–H in the range 0.95 Å–0.99 Å and *U*_iso_(H)=1.2*U*_eq_(C); (1.5 for H atoms of methyl groups).

### Crystal data

**Table:**

[FeCl_2_(C_18_H_25_N_3_O_2_)]	*F*(000) = 920
*M**_r_* = 442.16	*D*_x_ = 1.466 Mg m^−^^3^
Monoclinic, *P*2_1_/*c*	Mo *K*α radiation, λ = 0.71073 Å
Hall symbol: -P 2ybc	Cell parameters from 186 reflections
*a* = 10.102 (2) Å	θ = 3.3–21.8°
*b* = 13.925 (3) Å	µ = 1.04 mm^−^^1^
*c* = 14.764 (2) Å	*T* = 173 K
β = 105.27 (1)°	Prism, yellow
*V* = 2003.5 (6) Å^3^	0.20 × 0.10 × 0.10 mm
*Z* = 4	

### Data collection

**Table:**

Bruker–Nonius KappaCCD diffractometer	4578 independent reflections
Radiation source: fine-focus sealed tube	3033 reflections with *I* > 2σ(*I*)
Graphite monochromator	*R*_int_ = 0.054
Detector resolution: 9 pixels mm^-1^	θ_max_ = 27.5°, θ_min_ = 3.2°
CCD rotation images, thick slices scans	*h* = −12→13
Absorption correction: multi-scan (*SADABS*; Bruker, 2001)	*k* = −17→17
*T*_min_ = 0.820, *T*_max_ = 0.903	*l* = −17→19
16483 measured reflections	

### Refinement

**Table:**

Refinement on *F*^2^	Primary atom site location: structure-invariant direct methods
Least-squares matrix: full	Secondary atom site location: difference Fourier map
*R*[*F*^2^ > 2σ(*F*^2^)] = 0.041	Hydrogen site location: inferred from neighbouring sites
*wR*(*F*^2^) = 0.083	H-atom parameters constrained
*S* = 1.01	*w* = 1/[σ^2^(*F*_o_^2^) + (0.0268*P*)^2^ + 1.0658*P*] where *P* = (*F*_o_^2^ + 2*F*_c_^2^)/3
4578 reflections	(Δ/σ)_max_ = 0.001
240 parameters	Δρ_max_ = 0.34 e Å^−^^3^
0 restraints	Δρ_min_ = −0.30 e Å^−^^3^

### Special details

**Table:**

Geometry. All e.s.d.'s (except the e.s.d. in the dihedral angle between two l.s. planes) are estimated using the full covariance matrix. The cell e.s.d.'s are taken into account individually in the estimation of e.s.d.'s in distances, angles and torsion angles; correlations between e.s.d.'s in cell parameters are only used when they are defined by crystal symmetry. An approximate (isotropic) treatment of cell e.s.d.'s is used for estimating e.s.d.'s involving l.s. planes.
Refinement. Refinement of *F*^2^ against ALL reflections. The weighted *R*-factor *wR* and goodness of fit *S* are based on *F*^2^, conventional *R*-factors *R* are based on *F*, with *F* set to zero for negative *F*^2^. The threshold expression of *F*^2^ > σ(*F*^2^) is used only for calculating *R*-factors(gt) *etc*. and is not relevant to the choice of reflections for refinement. *R*-factors based on *F*^2^ are statistically about twice as large as those based on *F*, and *R*- factors based on ALL data will be even larger.

### Fractional atomic coordinates and isotropic or equivalent isotropic displacement parameters (Å^2^)

**Table:**

	*x*	*y*	*z*	*U*_iso_*/*U*_eq_	
Fe1	0.89069 (4)	0.34069 (2)	0.30170 (2)	0.01876 (10)	
Cl1	0.87074 (7)	0.33484 (5)	0.14547 (4)	0.03170 (17)	
Cl2	1.10937 (7)	0.33479 (5)	0.39080 (5)	0.03539 (17)	
O1	0.58286 (18)	0.18564 (11)	0.38732 (12)	0.0238 (4)	
O2	0.59661 (18)	0.52345 (11)	0.36945 (12)	0.0245 (4)	
N1	0.7675 (2)	0.24265 (13)	0.34819 (13)	0.0172 (4)	
N3	0.7766 (2)	0.45103 (13)	0.33782 (13)	0.0177 (4)	
N2	0.5051 (2)	0.35734 (15)	0.19970 (14)	0.0257 (5)	
C1	0.7779 (3)	0.13552 (16)	0.34031 (16)	0.0193 (5)	
C2	0.6654 (3)	0.10073 (17)	0.38508 (19)	0.0267 (6)	
H2A	0.6096	0.0494	0.3468	0.027*	
H2B	0.7059	0.0759	0.4493	0.027*	
C3	0.6578 (3)	0.26111 (16)	0.37190 (16)	0.0177 (5)	
C4	0.6652 (3)	0.44283 (16)	0.36183 (15)	0.0169 (5)	
C5	0.6871 (3)	0.60246 (17)	0.36051 (19)	0.0268 (6)	
H5A	0.7304	0.6313	0.4225	0.027*	
H5B	0.6359	0.6529	0.3183	0.027*	
C6	0.7948 (3)	0.55571 (17)	0.31880 (18)	0.0244 (6)	
C7	0.7456 (3)	0.11024 (19)	0.23679 (17)	0.0309 (6)	
H7A	0.6525	0.1318	0.2052	0.031*	
H7B	0.7518	0.0405	0.2298	0.031*	
H7C	0.8116	0.1421	0.2086	0.031*	
C8	0.9184 (3)	0.10127 (19)	0.3938 (2)	0.0331 (7)	
H8B	0.9881	0.1317	0.3680	0.033*	
H8A	0.9237	0.0314	0.3878	0.033*	
H8C	0.9348	0.1185	0.4602	0.033*	
C9	0.7624 (3)	0.57103 (19)	0.21363 (19)	0.0373 (7)	
H9B	0.8259	0.5331	0.1880	0.037*	
H9C	0.7726	0.6392	0.2005	0.037*	
H9A	0.6680	0.5507	0.1844	0.037*	
C10	0.9397 (3)	0.5868 (2)	0.3683 (2)	0.0381 (7)	
H10C	0.9567	0.5757	0.4359	0.038*	
H10B	0.9506	0.6553	0.3568	0.038*	
H10A	1.0053	0.5496	0.3441	0.038*	
C11	0.6263 (3)	0.36275 (18)	0.49971 (16)	0.0267 (6)	
H11A	0.5805	0.3094	0.5225	0.027*	
H11B	0.5905	0.4238	0.5163	0.027*	
H11C	0.7253	0.3594	0.5287	0.027*	
C12	0.4431 (3)	0.35778 (17)	0.34699 (16)	0.0213 (5)	
H12B	0.4009	0.3028	0.3715	0.021*	
H12A	0.4052	0.4174	0.3667	0.021*	
C13	0.5987 (2)	0.35583 (16)	0.39150 (15)	0.0177 (5)	
C14	0.4740 (3)	0.35449 (17)	0.10478 (16)	0.0253 (6)	
H14	0.5460	0.3588	0.0745	0.025*	
C15	0.3403 (3)	0.34549 (18)	0.05079 (18)	0.0332 (7)	
H15	0.3206	0.3424	−0.0157	0.033*	
C16	0.2350 (3)	0.34100 (19)	0.09531 (19)	0.0355 (7)	
H16	0.1424	0.3349	0.0593	0.035*	
C17	0.2649 (3)	0.34528 (18)	0.18914 (18)	0.0263 (6)	
H17	0.1937	0.3430	0.2202	0.026*	
C18	0.4028 (3)	0.35314 (16)	0.24126 (16)	0.0225 (5)	

### Atomic displacement parameters (Å^2^)

**Table:**

	*U*^11^	*U*^22^	*U*^33^	*U*^12^	*U*^13^	*U*^23^
Fe1	0.0160 (2)	0.02093 (18)	0.02094 (18)	0.00010 (16)	0.00769 (14)	0.00049 (15)
Cl1	0.0378 (4)	0.0372 (4)	0.0234 (3)	0.0015 (3)	0.0140 (3)	0.0002 (3)
Cl2	0.0185 (4)	0.0517 (4)	0.0342 (3)	0.0017 (3)	0.0038 (3)	0.0017 (3)
O1	0.0232 (11)	0.0157 (9)	0.0363 (10)	−0.0017 (7)	0.0145 (8)	0.0011 (7)
O2	0.0225 (11)	0.0166 (9)	0.0380 (10)	0.0018 (7)	0.0141 (8)	0.0010 (8)
N1	0.0174 (12)	0.0160 (10)	0.0184 (10)	0.0008 (8)	0.0048 (9)	−0.0005 (8)
N3	0.0167 (12)	0.0157 (10)	0.0215 (10)	−0.0015 (8)	0.0063 (9)	0.0002 (8)
N2	0.0259 (13)	0.0276 (12)	0.0242 (11)	0.0005 (10)	0.0076 (9)	−0.0008 (9)
C1	0.0208 (15)	0.0156 (12)	0.0207 (12)	0.0002 (10)	0.0045 (10)	−0.0012 (10)
C2	0.0315 (17)	0.0166 (13)	0.0353 (15)	−0.0001 (11)	0.0148 (12)	0.0009 (11)
C3	0.0171 (14)	0.0187 (12)	0.0166 (12)	−0.0017 (10)	0.0033 (10)	0.0024 (10)
C4	0.0181 (14)	0.0161 (12)	0.0158 (11)	0.0008 (10)	0.0033 (10)	−0.0013 (10)
C5	0.0275 (17)	0.0151 (13)	0.0398 (15)	−0.0049 (11)	0.0123 (13)	−0.0007 (11)
C6	0.0264 (16)	0.0152 (13)	0.0338 (14)	−0.0021 (11)	0.0118 (12)	0.0006 (11)
C7	0.0393 (19)	0.0257 (14)	0.0288 (14)	−0.0030 (13)	0.0112 (13)	−0.0042 (12)
C8	0.0264 (17)	0.0239 (14)	0.0455 (17)	0.0023 (12)	0.0033 (13)	−0.0018 (13)
C9	0.054 (2)	0.0259 (15)	0.0365 (16)	0.0012 (14)	0.0201 (15)	0.0082 (13)
C10	0.0278 (18)	0.0258 (15)	0.062 (2)	−0.0077 (13)	0.0142 (15)	−0.0048 (14)
C11	0.0331 (17)	0.0281 (15)	0.0203 (12)	0.0014 (12)	0.0098 (11)	0.0004 (11)
C12	0.0181 (14)	0.0210 (13)	0.0257 (13)	−0.0014 (10)	0.0076 (10)	0.0008 (11)
C13	0.0174 (13)	0.0191 (12)	0.0186 (11)	0.0002 (10)	0.0083 (10)	−0.0001 (10)
C14	0.0310 (16)	0.0229 (13)	0.0224 (13)	0.0020 (11)	0.0078 (11)	−0.0033 (11)
C15	0.052 (2)	0.0222 (13)	0.0200 (13)	0.0049 (14)	−0.0001 (12)	−0.0018 (12)
C16	0.0263 (16)	0.0253 (14)	0.0426 (16)	0.0021 (13)	−0.0124 (13)	−0.0020 (13)
C17	0.0216 (15)	0.0190 (13)	0.0386 (15)	0.0014 (11)	0.0082 (12)	−0.0056 (12)
C18	0.0235 (15)	0.0163 (12)	0.0268 (13)	0.0020 (11)	0.0052 (11)	−0.0004 (10)

### Geometric parameters (Å, º)

**Table:**

Fe1—N1	2.0821 (19)	C7—H7B	0.9800
Fe1—N3	2.073 (2)	C7—H7C	0.9800
Fe1—Cl1	2.2633 (7)	C8—H8B	0.9800
Fe1—Cl2	2.2552 (9)	C8—H8A	0.9800
O1—C3	1.349 (3)	C8—H8C	0.9800
O1—C2	1.453 (3)	C9—H9B	0.9800
O2—C4	1.340 (3)	C9—H9C	0.9800
O2—C5	1.458 (3)	C9—H9A	0.9800
N1—C3	1.272 (3)	C10—H10C	0.9800
N1—C1	1.502 (3)	C10—H10B	0.9800
N3—C4	1.271 (3)	C10—H10A	0.9800
N3—C6	1.505 (3)	C11—C13	1.551 (3)
N2—C18	1.335 (3)	C11—H11A	0.9800
N2—C14	1.354 (3)	C11—H11B	0.9800
C1—C8	1.508 (4)	C11—H11C	0.9800
C1—C7	1.518 (3)	C12—C18	1.507 (3)
C1—C2	1.536 (3)	C12—C13	1.535 (3)
C2—H2A	0.9900	C12—H12B	0.9900
C2—H2B	0.9900	C12—H12A	0.9900
C3—C13	1.507 (3)	C14—C15	1.381 (4)
C4—C13	1.506 (3)	C14—H14	0.9500
C5—C6	1.530 (3)	C15—C16	1.392 (4)
C5—H5A	0.9900	C15—H15	0.9500
C5—H5B	0.9900	C16—C17	1.339 (4)
C6—C9	1.515 (4)	C16—H16	0.9500
C6—C10	1.517 (4)	C17—C18	1.408 (4)
C7—H7A	0.9800	C17—H17	0.9500
			
N3—Fe1—N1	88.85 (7)	C1—C8—H8B	109.5
N3—Fe1—Cl2	113.73 (6)	C1—C8—H8A	109.5
N1—Fe1—Cl2	111.27 (6)	H8B—C8—H8A	109.5
N3—Fe1—Cl1	112.50 (6)	C1—C8—H8C	109.5
N1—Fe1—Cl1	114.32 (6)	H8B—C8—H8C	109.5
Cl2—Fe1—Cl1	113.81 (3)	H8A—C8—H8C	109.5
C3—O1—C2	106.19 (18)	C6—C9—H9B	109.5
C4—O2—C5	105.92 (18)	C6—C9—H9C	109.5
C3—N1—C1	107.81 (19)	H9B—C9—H9C	109.5
C3—N1—Fe1	126.70 (16)	C6—C9—H9A	109.5
C1—N1—Fe1	124.42 (15)	H9B—C9—H9A	109.5
C4—N3—C6	107.4 (2)	H9C—C9—H9A	109.5
C4—N3—Fe1	126.74 (16)	C6—C10—H10C	109.5
C6—N3—Fe1	124.51 (15)	C6—C10—H10B	109.5
C18—N2—C14	118.6 (2)	H10C—C10—H10B	109.5
N1—C1—C8	110.4 (2)	C6—C10—H10A	109.5
N1—C1—C7	107.91 (19)	H10C—C10—H10A	109.5
C8—C1—C7	112.1 (2)	H10B—C10—H10A	109.5
N1—C1—C2	101.83 (18)	C13—C11—H11A	109.5
C8—C1—C2	112.1 (2)	C13—C11—H11B	109.5
C7—C1—C2	111.9 (2)	H11A—C11—H11B	109.5
O1—C2—C1	104.33 (18)	C13—C11—H11C	109.5
O1—C2—H2A	110.9	H11A—C11—H11C	109.5
C1—C2—H2A	110.9	H11B—C11—H11C	109.5
O1—C2—H2B	110.9	C18—C12—C13	114.2 (2)
C1—C2—H2B	110.9	C18—C12—H12B	108.7
H2A—C2—H2B	108.9	C13—C12—H12B	108.7
N1—C3—O1	117.2 (2)	C18—C12—H12A	108.7
N1—C3—C13	130.4 (2)	C13—C12—H12A	108.7
O1—C3—C13	112.4 (2)	H12B—C12—H12A	107.6
N3—C4—O2	117.6 (2)	C4—C13—C3	114.71 (19)
N3—C4—C13	130.5 (2)	C4—C13—C12	110.27 (19)
O2—C4—C13	111.7 (2)	C3—C13—C12	110.26 (19)
O2—C5—C6	104.15 (18)	C4—C13—C11	106.07 (19)
O2—C5—H5A	110.9	C3—C13—C11	106.22 (19)
C6—C5—H5A	110.9	C12—C13—C11	109.02 (19)
O2—C5—H5B	110.9	N2—C14—C15	121.7 (2)
C6—C5—H5B	110.9	N2—C14—H14	119.2
H5A—C5—H5B	108.9	C15—C14—H14	119.2
N3—C6—C9	108.9 (2)	C14—C15—C16	119.0 (2)
N3—C6—C10	109.7 (2)	C14—C15—H15	120.5
C9—C6—C10	111.9 (2)	C16—C15—H15	120.5
N3—C6—C5	101.67 (19)	C17—C16—C15	119.7 (3)
C9—C6—C5	111.6 (2)	C17—C16—H16	120.2
C10—C6—C5	112.5 (2)	C15—C16—H16	120.2
C1—C7—H7A	109.5	C16—C17—C18	119.3 (3)
C1—C7—H7B	109.5	C16—C17—H17	120.3
H7A—C7—H7B	109.5	C18—C17—H17	120.3
C1—C7—H7C	109.5	N2—C18—C17	121.8 (2)
H7A—C7—H7C	109.5	N2—C18—C12	116.3 (2)
H7B—C7—H7C	109.5	C17—C18—C12	121.9 (2)
			
N3—Fe1—N1—C3	9.1 (2)	C4—N3—C6—C9	−105.0 (2)
Cl2—Fe1—N1—C3	124.2 (2)	Fe1—N3—C6—C9	62.7 (3)
Cl1—Fe1—N1—C3	−105.2 (2)	C4—N3—C6—C10	132.2 (2)
N3—Fe1—N1—C1	175.79 (18)	Fe1—N3—C6—C10	−60.1 (3)
Cl2—Fe1—N1—C1	−69.11 (18)	C4—N3—C6—C5	12.9 (3)
Cl1—Fe1—N1—C1	61.53 (18)	Fe1—N3—C6—C5	−179.43 (15)
N1—Fe1—N3—C4	−9.9 (2)	O2—C5—C6—N3	−17.1 (3)
Cl2—Fe1—N3—C4	−122.71 (19)	O2—C5—C6—C9	98.8 (2)
Cl1—Fe1—N3—C4	106.0 (2)	O2—C5—C6—C10	−134.4 (2)
N1—Fe1—N3—C6	−175.18 (19)	N3—C4—C13—C3	−13.5 (4)
Cl2—Fe1—N3—C6	72.03 (19)	O2—C4—C13—C3	171.6 (2)
Cl1—Fe1—N3—C6	−59.23 (19)	N3—C4—C13—C12	−138.7 (3)
C3—N1—C1—C8	−130.6 (2)	O2—C4—C13—C12	46.5 (2)
Fe1—N1—C1—C8	60.6 (2)	N3—C4—C13—C11	103.4 (3)
C3—N1—C1—C7	106.6 (2)	O2—C4—C13—C11	−71.4 (2)
Fe1—N1—C1—C7	−62.3 (2)	N1—C3—C13—C4	12.4 (4)
C3—N1—C1—C2	−11.4 (3)	O1—C3—C13—C4	−171.2 (2)
Fe1—N1—C1—C2	179.80 (15)	N1—C3—C13—C12	137.6 (3)
C3—O1—C2—C1	−14.9 (2)	O1—C3—C13—C12	−46.0 (2)
N1—C1—C2—O1	15.6 (2)	N1—C3—C13—C11	−104.4 (3)
C8—C1—C2—O1	133.6 (2)	O1—C3—C13—C11	72.0 (2)
C7—C1—C2—O1	−99.4 (2)	C18—C12—C13—C4	63.0 (2)
C1—N1—C3—O1	2.4 (3)	C18—C12—C13—C3	−64.7 (2)
Fe1—N1—C3—O1	170.87 (14)	C18—C12—C13—C11	179.02 (19)
C1—N1—C3—C13	178.6 (2)	C18—N2—C14—C15	−1.3 (4)
Fe1—N1—C3—C13	−12.9 (4)	N2—C14—C15—C16	1.2 (4)
C2—O1—C3—N1	8.5 (3)	C14—C15—C16—C17	−0.1 (4)
C2—O1—C3—C13	−168.4 (2)	C15—C16—C17—C18	−0.7 (4)
C6—N3—C4—O2	−3.3 (3)	C14—N2—C18—C17	0.4 (4)
Fe1—N3—C4—O2	−170.57 (15)	C14—N2—C18—C12	−179.3 (2)
C6—N3—C4—C13	−177.9 (2)	C16—C17—C18—N2	0.6 (4)
Fe1—N3—C4—C13	14.8 (4)	C16—C17—C18—C12	−179.7 (2)
C5—O2—C4—N3	−8.6 (3)	C13—C12—C18—N2	−6.2 (3)
C5—O2—C4—C13	166.98 (19)	C13—C12—C18—C17	174.1 (2)
C4—O2—C5—C6	16.0 (2)		
